# Balance and coordination after viewing stereoscopic 3D television

**DOI:** 10.1098/rsos.140522

**Published:** 2015-07-08

**Authors:** Jenny C. A. Read, Jennifer Simonotto, Iwo Bohr, Alan Godfrey, Brook Galna, Lynn Rochester, Tom V. Smulders

**Affiliations:** 1 Institute of Neuroscience, Newcastle University, Framlington Place, Newcastle upon Tyne NE2 4HH, UK; 2 Newcastle University, Clinical Ageing Research Unit, Campus for Ageing and Vitality, Newcastle upon Tyne NE4 5PL, UK

**Keywords:** stereoscopic vision, 3D displays, binocular vision, simulator sickness, binocular disparity

## Abstract

Manufacturers and the media have raised the possibility that viewing stereoscopic 3D television (S3D TV) may cause temporary disruption to balance and visuomotor coordination. We looked for evidence of such effects in a laboratory-based study. Four hundred and thirty-three people aged 4–82 years old carried out tests of balance and coordination before and after viewing an 80 min movie in either conventional 2D or stereoscopic 3D, while wearing two triaxial accelerometers. Accelerometry produced little evidence of any change in body motion associated with S3D TV. We found no evidence that viewing the movie in S3D causes a detectable impairment in balance or in visuomotor coordination.

## 1. Introduction

Stereoscopic 3D (S3D) displays are increasingly common. Every year, several major movies are released in 3D, and Blu-Ray technology means that they can be viewed in S3D at home. Most new television sets are now capable of displaying in S3D, and 3D TV channels are available in many countries around the world, including the USA, Europe and Japan. A minority of viewers report discomfort associated with S3D content [1–10]. The symptoms most commonly reported are headache and some form of visual fatigue or eyestrain. While these are annoying for viewers and certainly put people off 3D, they are not in themselves a serious concern. However, there have been undocumented suggestions of more serious concerns. Samsung, for example, supplies its 3D TVs with a leaflet entitled ‘Viewing TV using the 3D function: important safety information’. As well as warning that ‘Watching TV while wearing 3D glasses for an extended period of time may cause a headache or fatigue’, this document states that ‘Viewing 3D television may also cause motion sickness, perceptual after effects, disorientation, eye strain and decreased postural stability.’ It also implies that S3D may be associated with a range of other symptoms, including altered vision, dizziness, cramps, convulsions and loss of awareness. A report on S3D published by the French Agence nationale de sécurité sanitaire de l'alimentation, de l'environnement et du travail (Anses) warned that vertigo or alteration in visual perception caused by watching S3D could potentially lead to an increased risk of accident [11]. Unsurprisingly, such warnings have led to public concern, with government officials in Australia cautioning people about buying 3D televisions (http://www.abc.net.au/news/2010-06-14/consumers-warned-of-3d-tv-dangers/866572, retrieved 22 April 2013), or a newspaper in Britain suggesting that motion sickness caused by a S3D movie caused a driver to black out and crash his car on the way back from the cinema (http://www.dailymail.co.uk/health/article-1271618/How-watching-3D-films-bad-brain.html, retrieved 22 April 2013).

If viewing S3D really can cause dizziness, disorientation and decreased postural stability, people would run an increased risk of accident and injury after watching S3D content. It is clearly therefore of great public interest to establish whether this is the case, in order that appropriate risk management procedures can be put in place. In a previous paper [6], we reported that people who viewed 2D content while wearing 3D glasses reported dizziness at about the same rate as people viewing S3D content, also with glasses. This suggests that S3D itself does not cause dizziness; the dizziness may have been due to the 3D glasses [4] or to negative expectations. However, other effects such as headache and eyestrain were reported more often after viewing S3D [6]. It is possible in principle that these might be due to factors which could not only affect comfort, but also impact visuomotor performance. For example, viewing stereoscopic content produces measurable changes in accommodative response, i.e. how the eye focuses on objects at different distances [4,9]. In theory, if this impairment in accommodation persisted after viewing S3D content, it could cause a temporary reduction in visual acuity which could affect performance. Yet to our knowledge, there are no published studies which examine whether viewing S3D does produce measurable impairments on visuomotor tasks. The Anses report noted a lack of data in this area and urged research to explore the effects of exposure to S3D on the vestibular system, balance, postural control and gait [11].

In this study, we examined an exceptionally large number of participants, over 400 people ranging in age from 4 to 82 years. We measured performance on a range of visuomotor tasks, both before and after TV viewing. The tasks were designed to be safe to perform even if impaired, but to require good visuomotor performance. In this way, we aimed to reveal any impairments which would have an impact on everyday life.

## 2. Material and methods

Participants carried out a set of balance and coordination tasks, watched the animated Pixar movie ‘Toy Story’ in either 2D or S3D, and then repeated the balance and coordination tasks. These tasks are described in detail below. In total, 433 participants took part in the study, though not all data are available for all participants (table 1). One hundred and twenty-five of these participants went on to participate in a longer study [7]. We have reported on participants' subjective judgements about their viewing experience in a previous paper [6].

**Table 1.** Number of participants in each group. Subsequent rows show the number of participants for whom a complete set of coordination, balance and accelerometry data are available.

TV-group	A	B	C	D	E	totals
content viewed	active S3D	passive S3D	2D	2D	2D	
3D glasses worn	active S3D	passive S3D	none	active 3D, not shuttering	passive 3D	
number of participants	115	131	122	33	32	433
number with complete coordination data (before and after TV viewing, on same track)	113	125	119	33	30	420
number with complete balance task timing data (before and after TV viewing)	113	130	116	33	32	424
number with complete accelerometry data available (before and after TV viewing, hip and chest accelerometers)	107	120	111	26	25	389

### 2.1 TV-groups

Initially, participants were assigned in alternation into one of three groups, designated A, B and C. The ‘A’ group viewed an S3D movie presented on an active 3D TV set (LG model 47LX6900). The ‘B’ group viewed an S3D movie presented on a passive 3D TV set (LG model 47LD920). The ‘C’ group watched the same movie in 2D, as a control, and it was presented on the active 3D TV operated in its 2D mode. We did not ask these participants to wear 3D glasses while watching the 2D movie, as we did not want to tell them they were a control group, and we assumed they would object that the 3D was ‘not working’ if we asked them to wear 3D glasses while the content they were viewing was 2D. However, during the course of the study, we discovered that participants were quite happy to view 2D content while wearing 3D glasses. Apparently, the non-stereo depth cues present in the content, along with the context provided by the 3D glasses, convinced them that they were indeed viewing S3D. Thus, towards the end of the study, we introduced two further groups, which we designated ‘D’ and ‘E’. Both these watched the same 2D content as the C group. D-group participants wore the active 3D shutter glasses, although these were not shuttering at the time. E-group participants wore the passive 3D glasses. The D and E participants were drawn from the sample of late-entrants only, and have a somewhat different demographic profile, as described in [6]. The viewing conditions are also described in detail in [6]. Briefly, they were designed so as to simulate a living-room environment with a viewing distance of around 2.5 m.

### 2.2 Coordination tests

**Figure 1.**
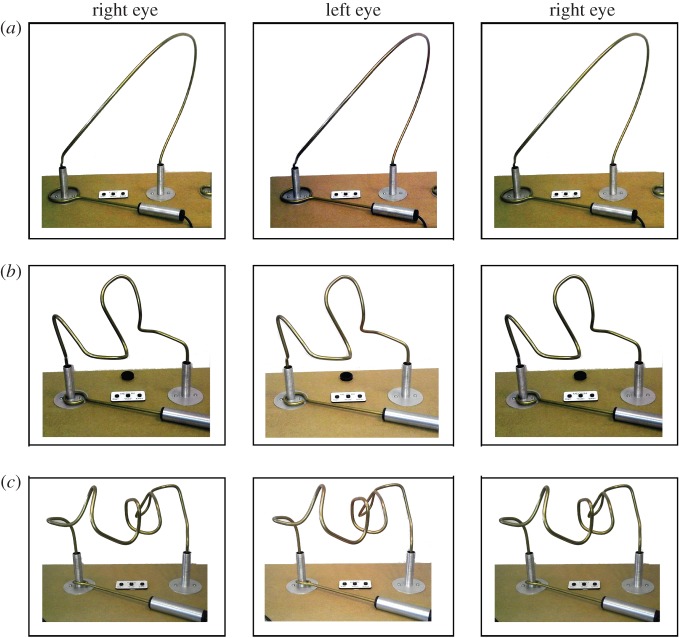
The coordination tests. Stereopairs showing the easy (*a*), medium (*b*) and hard (*c*) tests. The tests differed in the complexity of the track and in the diameter of the loop. The black borders are there to aid fusion. According to preference, the stereopairs can be viewed with crossed eyes such that the right eye views the middle column and the left eye views the leftmost column, or with parallel/diverged eyes such that the right eye views the rightmost column and the left eye views the middle column.

**Figure 2.**
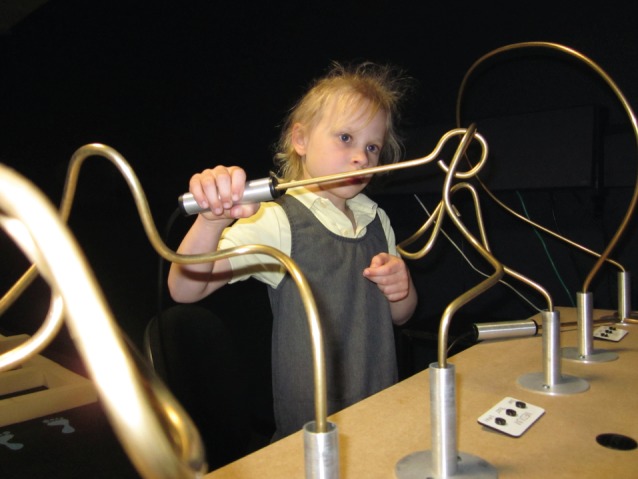
Child participant doing the coordination task (medium track).

Participants completed a visually guided manual dexterity task, which required them to guide a loop of wire around a wire track without touching the track (figures 1 and 2). Touches resulted in a buzzing sound and a red light-emitting diode lighting up. The wire track was non-planar, meaning that the loop had to be guided in depth as well as laterally and vertically, maximizing the demands on stereo vision [12]. This task has been proposed as a screening test for professions such as surgery which require high visuomotor skills [12], and good performance requires the use of both eyes; the error rate triples with monocular viewing [13]. The start and end times, and a voltage representing errors made during the task, were recorded using a data acquisition system from National Instruments (Austin, TX, USA), and automatically recorded by the computer running the experiment using code written in Matlab (The Mathworks Inc., Sherborn, MA, USA; www.mathworks.com).

Given the wide age range of the participants, the coordination task bench had three different wire tracks: easy, medium and hard. The aim was to ensure that every participant completed a task which was challenging for them, to avoid floor and ceiling effects. Thus, if a participant initially chose a track which they were able to complete without error, they were asked to do a harder task. In the analysis, we only compared values measured on the same track (before and after TV viewing). In a few cases where a participant did two tracks both before and after, we took values from the harder track.

### 2.3 Balance tests

**Figure 3.**
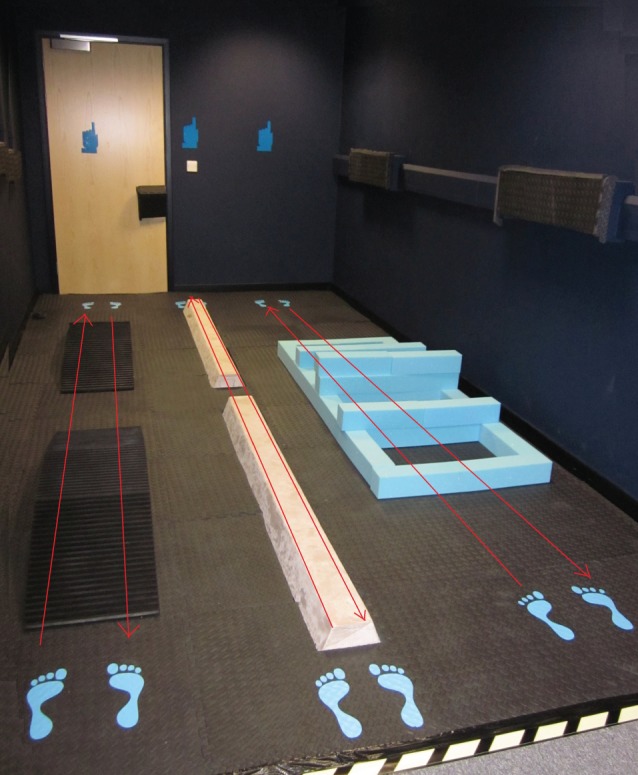
TV reporting room showing balance task. The balance task consists of three phases: ramp, beam and step. Arrows show the path participants took around the course.

To assess participants' balance and stability, we asked them to walk around the obstacle course shown in figure 3. This has three components: the ramp, beam and steps. In each case, participants simply walked along the obstacles to the far end of the room, turned around and retraced their steps over the obstacle. Pressure sensors under the foam floor mats recorded when they completed each section and the computer controlling the experiment automatically recorded this objective timing information to disc. Participants wore two triaxial accelerometers to monitor their movements during the task. Participants who felt unable to complete the obstacle course safely, e.g. due to mobility impairment, opted out of the balance tests.

#### 2.3.1 Ramp task

**Figure 4.**
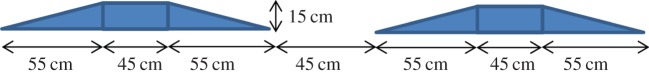
Dimensions of the ramp task as seen from the side. The ramps were 40 cm wide and made of black ridged plastic.

Participants walked over two identical ridged rubber ramps with the dimensions shown in figure 4. It was suggested by Helmholtz [14] that stereo vision is optimized for depth perception in the ground plane [15], and this task challenges participants to use their depth perception to calculate the location of this uneven surface and guide their feet appropriately. Impaired perception or balance would be expected to result in a slower time to complete this task, and/or a change in postural stability as estimated from the accelerometer data.

#### 2.3.2 Beam task

Participants walked along two identical foam beams, each 2 m long and a height of 8 cm above the foam floor mats and stepped over a 40 cm gap between the two beams. The beams were soft and yielded to the feet, making balance somewhat challenging. Pressure sensors under the floor recorded whether participants stepped off the beam, while the accelerometers worn at the hip and chest recorded body motion. Any dizziness or impaired balance should result in either a longer time to complete the task, or a greater likelihood of making an error, or greater body motion (wobble) during the task.

#### 2.3.3 Steps task

**Figure 5.**
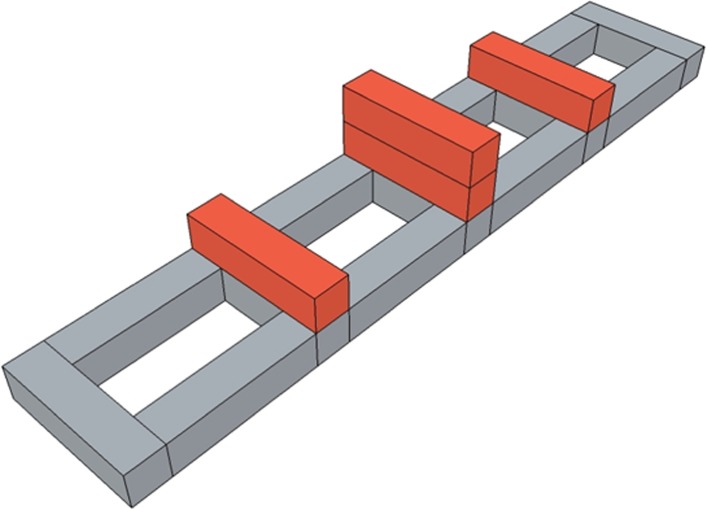
Arrangement of foam blocks in the steps task. The blocks shown in grey were glued together; those shown in red were loose and could be dislodged if participants knocked them as they stepped over them. The actual blocks were all the same colour, blue (figure 3).

Participants were asked to step through a grid formed by foam blocks, stepping in each of four spaces between the blocks (figure 5), without dislodging the blocks shown in red in figure 5, which were not secured but rested on top. This required accurate depth perception and the ability to guide the feet precisely. Impaired perception, balance or coordination could show up in either a longer time to complete the task, or a greater likelihood of dislodging a block, or greater body motion during the task.

### 2.4 Accelerometry

**Figure 6.**
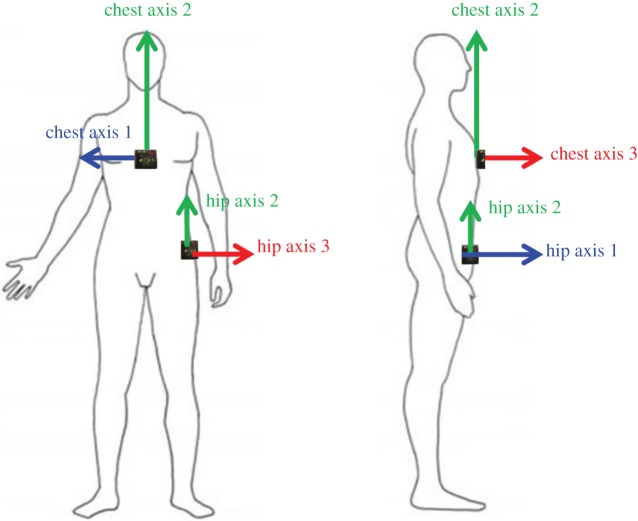
The accelerometer axes. The three axes along which accelerations are recorded, for the chest and hip accelerometers. Note that the accelerometers use a left-handed coordinate system. These directions are only approximate, since the precise direction will depend on the participant's body shape and precisely how they attached the accelerometer.

Participants wore two triaxial accelerometers, at hip and chest, throughout the experiment (figure 6). These were the AX3 logging sensor from Axivity, York, UK (axivity.com), with dimensions of 30×25×10 mm and weight of 12 g. We used the accelerometer data to extract a quantitative estimate of postural stability during the balance task.

**Figure 7.**
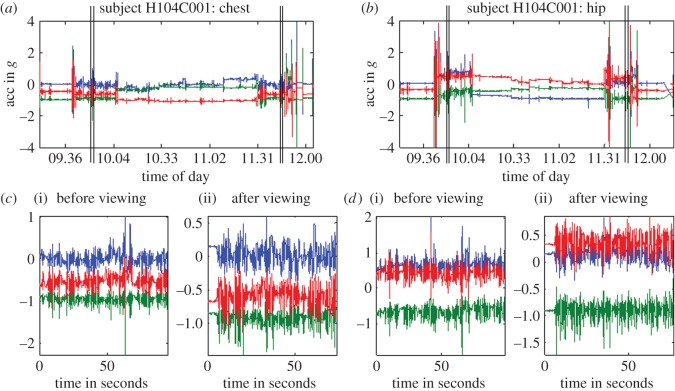
Example accelerometer data from one participant (unfiltered). The coloured traces show the acceleration recorded along each of three different axes, in units of g (10 m s^−2^); colours as in figure 6. Plots (*a*,*b*) show all data recorded. The vertical lines mark the time the participant performed the balance test before and after TV viewing. Plots (*c*,*d*) show the data during this period on a greatly expanded scale. Plots (*a*,*c*(i)(ii)) show data from the accelerometer on the participant's chest; (*b*,*d*(i)(ii)) from the accelerometer on their hip.

Each accelerometer constantly recorded the instantaneous accelerations along three axes, both accelerations due to the participant's body movement and the constant acceleration due to gravity. Figure 6 shows the approximate direction of each axis. Accelerations were recorded at 100 Hz and were clipped at ±4 g. Example accelerometry traces are shown in figure 7.

For analysis, the accelerometry data were passed through a second-order Butterworth filter with a high-frequency cut-off of 40 Hz to remove high-frequency noise while retaining moderate frequencies associated with postural instability and possible sudden movements. Figure 7*c*,*d* shows the accelerations recorded during all three balance tasks (ramp, beam and steps). For analysis, we divided the filtered accelerations recorded along each axis into separate traces recorded during each task. We then took all the numbers in each trace and calculated their standard deviation (s.d.). In this way, for each balance task, we converted our accelerometry data into a set of six numbers characterizing body movement [16,17].

### 2.5 Power calculation

We calculated the minimum effect size that our sample would have been likely to detect. In this study, we have measured changes in several different parameters, e.g. time taken to complete balance task, before and after TV viewing. In each case, our null hypothesis is that TV-group (3D-active, 3D-passive or 2D) has no effect on the change *x* under consideration, i.e. that the mean change *x* is the same for all three groups. We express the effect size in terms of *f*, where *f*^2^ is the ratio of the variance of the group means to the variance within the groups, assuming that the within-group standard deviation *σ* is the same in all groups [18]:

$$f^{2}=\frac{\mathrm{var}(m_{i})}{\sigma^{2}},\;\sigma^{2}=\mathrm{var}(x_{ij}-m_{i}),$$

where *i* indexes TV-group, *j* indexes participants; *n*_*i*_ is the number of participants in the *i*th group and m_*i*_ the mean in the *i*th group: $m_{i}=\sum_{j=1}^{n_{i}}x_{ij}\text{/}n_{i}.$

Given that the mean sample size in each of our five groups is 87, we would require an effect size of *f*>0.1 in order to achieve a power *π* of 0.8, i.e. to correctly reject the null hypothesis 80% of the time [18]. It will be helpful to express this as the smallest detectable spread in group means, as a function of the within-group standard deviation *σ*. Assuming intermediate variance between the different TV groups, the smallest range we could detect would be *f*√6=0.3 [18]. That is, our study has enough power for us to reliably detect an effect of 0.3 of the within-group standard deviations.

## 3. Results

### 3.1 Coordination task

Here, we have two basic performance metrics, corresponding to speed and to accuracy, respectively: the time taken to do the task, and the percentage of time spent in contact with the wire. Because participants could choose from three different tracks, it is not meaningful to compare results directly across participants. What is relevant is any change in performance after TV viewing, relative to the baseline performance beforehand. For each performance metric, therefore, we examined any change, both in absolute terms (e.g. how many seconds faster someone is) and in percentage terms (by what percentage their time decreases). For each participant, we only compared values measured on the same track. These data were available for 420 participants.

**Figure 8.**
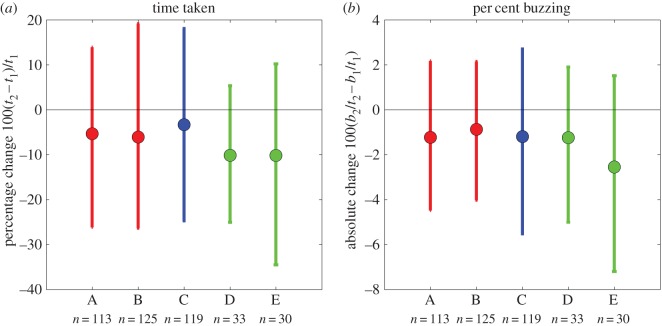
Improvements on the coordination task after viewing TV. Panel (*a*) examines changes in total time taken (*t*). Panel (*b*) examines changes in time spent buzzing (*b*). If *t*_1_ is the total time taken to complete the task before viewing TV and *b*_1_ is the time spent buzzing, and *t*_2_, *b*_2_ are the equivalent after viewing TV, then panel (*a*) plots percentage increase in time, 100(*t*_2_−*t*_1_)/*t*_1_, while panel (*b*) plots absolute change in percentage points of time spent buzzing, 100(*b*_2_/*t*_2_−*b*_1_/*t*_1_). We did not express this as a percentage change, because this could be infinite (*b*_1_ was zero for some participants). In both panels, negative values represent an improvement, i.e. less time taken/less time spent buzzing after viewing TV. Symbols represent the mean over all participants in the group for whom data were available (*n* shown below each group); error bars show the range from the 16th to 84th percentiles (equivalent to plotting ±1 s.d. for normally distributed data). Groups: A, active S3D; B, passive S3D; C, 2D control; D, E, ‘fake 3D’.

Figure 8 shows the mean changes for the five groups for both these tasks. Figure 8*a* shows percentage change in time taken. The means lie slightly below zero in each case, doubtless reflecting the effect of practice. This effect is small; on average people get about 6% faster and spend about 1% less time buzzing, but highly significant (*p*<10^−6^ for both metrics, *t*-test). However, there is no evidence that the type of TV viewed has any effect on performance. There are small differences between groups, but these are dwarfed by the variance between participants. Figure 8*b* shows the absolute change in per cent time spent buzzing. Again, we see a practice effect, but there is no difference between TV-groups compared to the variance between participants.

We explored several ways to assess the significance of these differences. A one-way ANOVA revealed no effect of TV-group on the change before versus after TV viewing (*p*>0.43 for both metrics in figure 8). We also used Monte Carlo resampling as a non-parametric technique. That is, we tested the null hypothesis that there are no differences between groups by combining all the values (i.e. the changes after–before) into a single set *S*. Then to generate a resampled dataset for a group with *N* participants, we randomly picked, with replacement, *N* values from this set and took their mean. We then asked, for each pair of groups, how often the absolute value of the difference in the resampled means exceeded the absolute value of the difference in the means of the actual data for these groups. The answer was never less than 5%, indicating that there were no significant differences between groups. This conclusion held whether we considered all five groups, or whether we grouped A and B participants into a single S3D group and compared them with the 2D participants in groups C, D and E. We conclude that viewing S3D content has no effect on performance on this demanding visually guided manual coordination task.

Several studies have suggested that impaired or absent stereo vision is associated with poorer performance on manual tasks [19–22]. Specifically on the buzz-wire task, Murdoch *et al.* [12] found that stereoblind participants performed substantially worse than those with normal stereoacuity. In a previous study using a buzz-wire task, we also found that people with below-median stereoacuity performed worse, but this was not significant [13]. In this study, we have stereoacuity for most participants on both the Frisby near stereo test and the FD2 distance stereo test, measured by qualified orthoptists [6]. To investigate whether there was a relationship between performance on the coordination task and stereoacuity, we examined the data before TV watching. Since here we are looking for differences between participants, rather than within participants, we restricted this analysis to the ‘medium’ track, where we had the most data: 345 participants completed the ‘medium’ track before viewing TV. We had stereoacuity data for 260 of these participants. Defining ‘poor’ stereoacuity as an FD2 stereothreshold of 50 arcmin or higher, the six participants with ‘poor’ stereoacuity actually completed the task slightly faster than the 254 participants with ‘good’ stereoacuity (42±5 s versus 52±1 s, mean time ± s.e.), but they made slightly more errors (percentage time spent buzzing was 3.3±0.7% compared to 2.6±0.2%). However, these differences were not significant under bootstrap resampling. Similarly, if we define ‘poor’ stereoacuity as a Frisby stereothreshold of more than 200 arcmin, then five participants have ‘poor’ stereoacuity, including four of the six classed as ‘poor’ on the FD2. Again, these participants have a slightly higher error rate, but the difference is not significant. We have investigated other possible boundaries for ‘poor’ stereoacuity and examined the correlation between stereo threshold and performance metrics, but have not been able to identify any significant relationship between stereoacuity and performance on the coordination task. It is disappointing that we have failed in two separate studies to reproduce the finding of Murdoch *et al*. that stereoblind individuals have a significantly higher error rate on this task. Possible reasons include the fact that they recruited a high percentage of stereoblind participants (17/71 or 24%); in our study, only 4/334 (1%) participants were stereoblind on the Frisby test and only 9/331 (3%) on the FD2 (i.e. could not do the task at any disparity despite demonstrating understanding of the test). If Murdoch *et al.* [12] are correct that only relatively crude stereo vision is required to perform this task and thus that only truly stereoblind individuals are impaired, we may simply not have had enough stereoblind participants for the difference to reach significance.

### 3.2 Balance tasks

#### 3.2.1 Performance metrics

As for the coordination task, performance on the balance tasks can be assessed both by accuracy and by speed. The accuracy metrics were, first, whether or not the participant managed to walk along the beam without stepping off it, and second, the number of foam blocks they dislodged from the steps while attempting to step over them. The speed metrics were the times taken to do the task, and each of its subcomponents (ramp, beam and steps), recorded automatically by pressure-sensitive mats under the floor. In each case, what is critical is not so much the performance of any individual participant, but any change in performance as a result of watching TV. This within-subjects design removes much of the noise due to individual variation. Data from the balance task were available for 424 participants.

**Figure 9.**
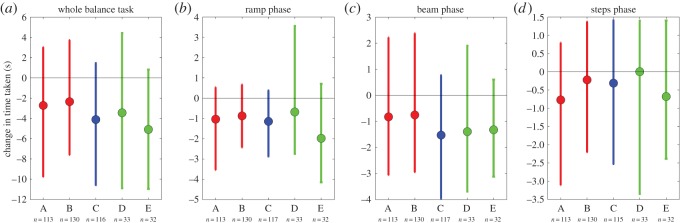
Change in time taken on (*a*) the entire balance task, and (*b*–*d*) its different components. Within-subjects mean of time-after minus time-before TV viewing, so negative numbers represent an improvement in performance after TV viewing. The horizontal line marks zero, i.e. no change. The differences between groups were not significant. Symbols show mean and error bars show the range from the 16th to 84th percentiles.

We first examined the change in time taken on the whole balance task (figure 9*a*) and on the three different components (ramp, beam and steps; figure 9*b*–*d*). In each case, we subtracted each individual's time *before* TV viewing from their time *after* TV viewing, to get the change for that individual. Figure 9 shows the mean value of these changes for participants in each of the five TV-groups. Participants in all groups are about 1 s faster on each component of the balance task after viewing TV, resulting in an overall speed-up of about 3 s on the whole balance task. As in the coordination task, figure 8, this is probably a practice effect. However, there is no effect of S3D versus 2D TV. We examined the differences between the groups using one-way ANOVA with TV-group as the factor, as well as bootstrap resampling the pairwise contrasts between individual TV-groups and between all 3D groups versus all 2D groups. None of the differences were significant.

**Figure 10.**
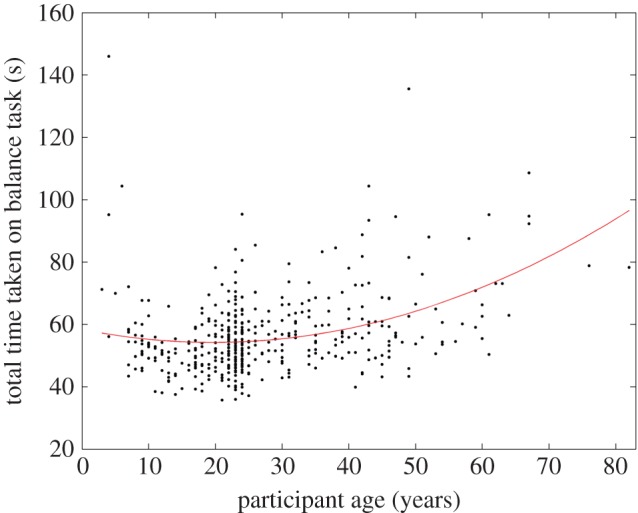
Time taken to complete the balance task, as a function of age. The red line shows a parabola fitted to the data with the Matlab function polyfit: time= 58.4−0.43 age+0.01 age^2^. The last two coefficients are significantly different from zero.

In contrast to TV-group, we do pick up an effect of age. Figure 10 shows time taken to complete the whole balance task, as a function of age. The red line is a parabolic fit. We see that the youngest participants are slowest, then speed improves until about 20 years old, then slowly declines with age. This is significant; the coefficients describing the dependence on age lie outside 95% confidence intervals generated by fitting parabolas to scrambled data, in which the ages have been shuffled randomly. Thus, it is not the case that our data are simply too noisy for us to detect any effects.

We next examined the probability of stepping off the beam, before versus after TV viewing. Only around 10% of participants stepped off the beam while performing the task, so the numbers involved were small and subject to large statistical fluctuations. There were no significant differences between the three different groups, nor between before versus after viewing.

**Figure 11.**
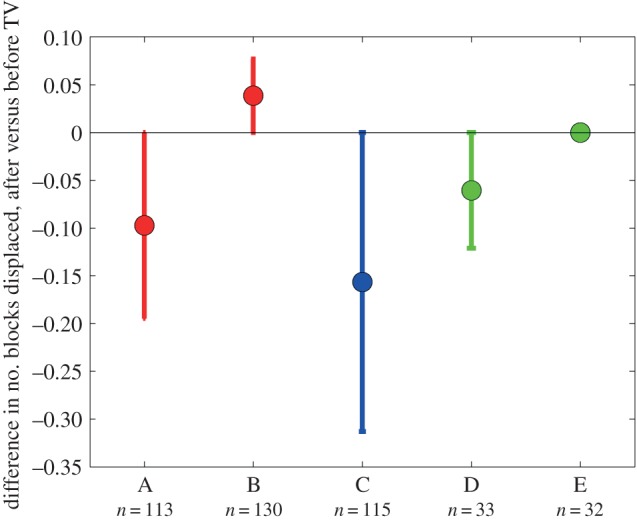
Change in number of blocks displaced on the steps task (figure 5). For each participant, we subtracted the number of blocks displaced before TV viewing from the number displaced after TV viewing. The data points show the mean of this difference for participants in the five TV-groups; error bars show the range from the 16th to 84th percentiles.

On the steps task, we examined how likely people were to knock over one of the foam blocks. Figure 11 shows the change in the number of blocks displaced, before versus after TV viewing. Again, negative numbers represent an improvement: fewer blocks displaced after TV viewing. Most groups do show this improvement. The B-group, who watched passive 3D TV, is slightly worse after viewing. However, this difference is not significant under one-way ANOVA, nor under bootstrap resampling.

#### 3.2.2 Accelerometry

We next turn to the accelerometry data. For this, we ran a mixed-design ANOVA with three within-subject factors: ‘axis’ (the six axes recorded from the two triaxial accelerometers), ‘task’ (ramp, beam and step) and ‘session’ (before versus after watching the film); and two between-subject factors: ‘TV-group’ (the five groups A, B, C, D and E) and ‘age-group’. For the last factor, we grouped participants into four groups based on their age: under 11 (*N*=74); 11–29 (*N*=65); 30–40 (*N*=257); over 41 (*N*=37). (N.B. Participants only reported year of birth, so strictly the above age-groups refer to those born after 2000, those born 1982–2000, etc; the year of testing was 2011.) The dependent variable was the s.d. of the filtered accelerometry trace specified by the value of ‘axis’. This is a measurement of the variability in body motion.

First, we demonstrate that our accelerometry is sensitive enough to detect differences in body motion during the different components of the balance task. The s.d. of the signal is largest while doing the steps task, then the ramp and smallest for the beam (task: *F*_2,361_=40.651,*p*<0.001). The s.d. of the signal differed for the different accelerometer axes (axis: *F*_5,358_=45.439,*p*<0.001), and as expected from the different body movements required for the three tasks, the effect of task was different for the different axes (axis × task interaction: *F*_10,353_=33.204,*p*<0.001).

**Figure 12.**
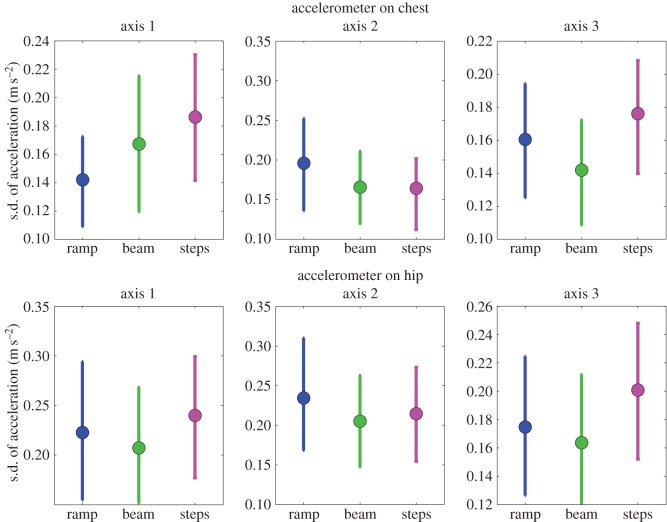
Mean s.d. of acceleration recorded during the different tasks. For each participant, we used the times recorded by the floor pressure sensors to extract the accelerometry data recorded while they were performing each phase of the balance task. Because each participant was wearing two triaxial accelerometers, this produced six sets of data, shown in the six panels of the figure. We filtered the accelerations with a 40 Hz cut-off, and then calculated the s.d. of each set of filtered accelerations. We did this for each participant, both before and after they watched TV. We then averaged in order to obtain the mean s.d. values shown in the figure. For this figure and the one-way ANOVA reported in the text, we pooled across TV-groups and across before versus after viewing. This means that each data point in the figure is the mean of around 800 separate s.d. values.

This is illustrated in figure 12. The different panels show the six different accelerometry axes. In each panel, the three data points show the standard deviation of these accelerations during the different balance tasks. In all six of these panels, one-way ANOVA reports a highly significant effect of task. Thus, even this simplest of analyses is enough to reveal the different body motion of participants on the three different balance tasks.

After viewing the movie, participants were led to a separate reporting room where they were asked about their subjective experience [6]. They were first asked to give a 7-point Likert rating in answer to the questions ‘How would you rate the visual appearance of what you watched today?’ and ‘Specifically, how realistic did you find the 3D depth?’. They were then asked ‘Did you experience any unpleasant effects or sensations?’. Participants who answered ‘yes’ to this were invited to pick the relevant effects from a list including impaired coordination or balance; they could also specify their own answers. These subjective reports are analysed in a separate publication [6].

A total of 66/433 participants reported any adverse effects. Only nine participants reported feeling dizzy or faint. These nine participants ranged in age from 18 to 35; they were made up of five A-group (active 3D), two B-group (passive 3D), one D-group (2D viewed with active 3D glasses) and one E-group (2D viewed with passive 3D glasses). Thus, all believed they were watching 3D. As we and others have reported elsewhere [1–3,6–10,23–27], S3D TV does seem to be associated with such subjective side-effects in a minority of participants, although most commonly headache and eyestrain. Dizziness/faintness is a rare side-effect and may be due to negative expectations surrounding 3D [6]. We examined our accelerometry data to see whether we could detect an objective effect in these nine participants, compared to the 367 (85%) who reported no adverse effects. For example, we looked to see whether their accelerations were more variable, reflecting reduced postural stability. However, no such difference was detectable. Overall, we found no relationship between change in acceleration variability and whether or not participants reported adverse effects (if we analyse the six axes separately, *p*>0.13 for each axis, Wilcoxon rank sum comparing change in standard deviations recorded during the entire balance task, for participants who reported adverse effects versus those who did not; if we combine data recorded from all six axes, *p*=0.35, Wilcoxon rank sum).

Overall, young children (age-group 1) showed higher variability than the older participants, with the teens/young adults (age-group 2) sitting between the young children and the older groups (*F*_3,362_=14.667, *p*<0.001). There were no differences between the two older groups. This age effect was stronger for the ramp task, where age-group 2 was also significantly more variable than the two adult groups (but less than the children), whereas in the other two tasks, age-group 2 was not significantly different from the adults (age × task interaction: *F*_6,724_=6.935, *p*<0.001). We also found that people in the different age-groups moved differently, as indicated by the fact that the differences in signal s.d. between the different axes depended on the age of the participants (age × axis interaction: *F*_15,1080_=4.303, *p*<0.001).

For all participants, the variability of the accelerometry traces increased significantly after watching the film (*F*_1,362_=14.646, *p*<0.001). This was independent of TV-group (session × TV-group interaction: *F*_4,362_=1.938, *p*=0.104) or age-group (session × age-group interaction: *F*_3,362_=0.640,*p*=0.589). Participants in group A (active 3D) had higher variability than the other groups (*F*_4,362_ = 7.180,*p* < 0.001), independent of whether they had watched TV yet, indicating that our randomization procedures in assigning participants to conditions had not worked as well as hoped. Indeed, analysing the data based on the first session alone, i.e. before TV viewing, reveals the same effect (*F*_4,364_=5.378,*p*<0.001). Similarly, we found a small effect of TV-group on which axis detects more variation (axis × TV-group interaction: *F*_20,1444_=1.723,*p*=0.024), but again, this is unrelated to the actual viewing of the film (axis × TV-group × session interaction: *F*_20,1444_=0.913,*p*=0.570) and indicates a failure of perfect randomized allocation to the groups.

**Figure 13.**
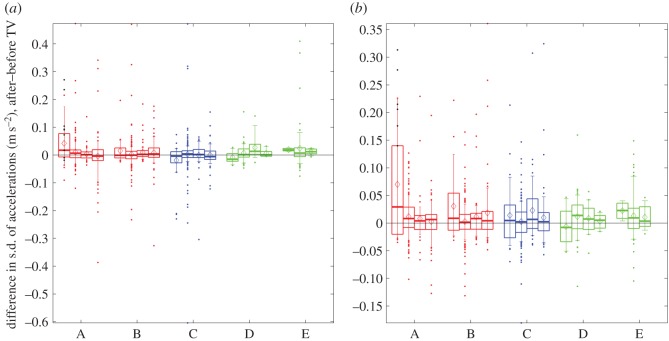
Change in variability of body motion during the balance test for (*a*) chest accelerometer and (*b*) hip accelerometer. The figure shows box-and-whisker plots for the change in standard deviation of acceleration, after TV viewing compared to before. For this figure, we have calculated the s.d. over the entire accelerometer trace recorded during the balance test, not divided up by task as for the analysis. We have also pooled s.d. changes from all three axes. Horizontal lines show the median; diamonds the mean. Boxes show the interquartile range; error bars link the 9th and 91st percentiles; coloured dots show outliers beyond this range. The four boxes for each TV-group show the four age-groups. TV-group E had no participants in age-group 4. Black dots show differences in s.d. for the three participants in age-group 1 who reported adverse effects (H222A004, H225A003 and H225A004, discussed in the text). There are three dots for each participant, reflecting the three axes. Hip accelerometry was not recorded for H225A003, so there are only six black dots in (*b*).

We did find a significant three-way interaction (session × TV-group × age-group: *F*_11,362_=2.717, *p*=0.002), which was caused by the fact that variability increased significantly more for the youngest participants in TV-group A than for any other participants. This is shown in figure 13, which plots the change in variability (s.d. after–s.d. before TV) for the different TV- and age-groups. The change is greater for the youngest age-group in TV-group A on both accelerometers (12 participants), and for the youngest age-group in TV-group B on the hip accelerometer (eight participants).

However, the significance of this interaction is largely driven by just two children, participants H222A004 and H225A004. These were two of the three (out of 37) children in age-group 1 who reported experiencing adverse effects. All three were in group A, active 3D; the third, H225A003, was the brother of H225A004, but due to a technical failure, hip accelerometry was not available for H225A003, so his data could not be used in the ANOVA analysis reported above. Participant H222A004, aged 9, reported eyestrain; the siblings H225A003 and H225A004, aged 8 and 5, reported eyestrain and nausea, respectively. All three children had specific issues. Participant H222A004 had recently been prescribed reading glasses for accommodative esotropia, i.e. to prevent crossing of the eyes caused by long-sightedness. We did not record whether she wore these during TV viewing. H225A003 also had esophoria, i.e. a tendency for his eyes to turn inwards (12 prism dioptres, prism cover test at 33 cm). The research assistant recorded that both H225A003 and H225A004 took off their 3D glasses at some point during the movie. This means that for an unknown duration, these participants were viewing blurred 2D television rather than S3D. No other participants were recorded by the research assistants as having removed their glasses.

As shown by the black dots in figure 13, these three participants showed the highest increases in variability for their age-group. The changes in variability for these three participants were significantly different from those recorded for their peers who did not report adverse effects (*p*<10^−5^, Wilcoxon rank-sum test, combining data from all available axes). When the two participants H222A004 and H225A004 are removed from the analysis, the interaction with TV-group is no longer significant (session × TV-group × age-group interaction now *F*_11,360_=1.496, *p*=0.131). This means that there is then no significant interaction term including TV-group and session, i.e. no result indicating a possible effect of TV viewing upon body motion.

### 3.3 Power of the study

In the Material and methods, we calculated that our sample size enabled us to detect a difference between TV-groups of about 0.3 within-group standard deviations. Table 2 shows what this means in practice for some of the parameters we measure. For example, consider error rate on the coordination task, defined as percentage of time on the task spent buzzing. Participants were on average 1.2 percentage points more accurate when tested for the second time, presumably due to practice. As shown in figure 8*b*, there was no difference between TV-groups in this improvement. We estimate we could have detected a difference of around 1.3 percentage points between groups. For example, if S3D impaired coordination, such that the S3D groups did not improve, whereas the 2D groups improved by 2 percentage points, we would have detected that. Our study does not allow us to rule out a smaller difference, e.g. say a difference of 1 percentage point between S3D and 2D groups.

**Table 2.** Minimum detectable effect sizes in our study. Each row shows a different quantity measured in our study. The third column shows the mean change in this quantity, subtracting the value measured before TV viewing from that measured after, averaged over all participants for whom the measurement was available. The fourth column shows 0.7 times the within-group standard deviation, which is the smallest difference in this change we would expect to be able to detect.

change in: (after–before)	units	mean value of change	minimum detectable difference in change
time taken to complete coordination task	seconds	−2.9	4.3
time spent buzzing on coordination task	seconds	−0.78	0.9
percentage of time spent buzzing	% points	−1.2	1.3
time taken to complete all three balance tests	seconds	−3.2	2.7
time taken to complete ramp task	seconds	−1.1	0.9
time taken to complete beam task	seconds	−1.1	1.2
time taken to complete steps task	seconds	−0.4	1.1
number of blocks displaced		−0.06	0.2
chest accelerometer: standard deviation of accelerations recorded during all three balance tests, mean over the three axes	m s^−2^	0.06	0.16
chest accelerometer: standard deviation of accelerations recorded during all three balance tests, mean over the three axes	m s^−2^	0.10	0.12

## 4. Discussion

In some viewers, certain S3D content can produce adverse effects such as eyestrain, headache or dizziness [2,28]. The reason for these effects is not yet entirely clear. In principle, vergence/accommodation conflict can cause eyestrain [1,8,10,24,25,27,29–32], but the magnitude of this conflict is deliberately kept low in commercial S3D content. The conflict between on-screen cues indicating observer motion and vestibular cues indicating that the observer is stationary can cause dizziness and nausea in susceptible individuals [33]. This conflict also exists in conventional 2D content, but since most viewers are more familiar with 2D content, they may have learnt to discount the conflict in this case [34]. It has been suggested that S3D may also cause changes in postural stability and perception of the environment which could potentially lead to an increased risk of accident [11].

This study addresses this issue by examining performance on a set of tasks designed to probe balance and coordination, both before and after participants viewed a movie in either 2D or S3D. We used objective performance metrics such as time taken to complete the tasks, error rate and body motion measured with triaxial accelerometers. In general, participants performed slightly better after TV viewing, doubtless a simple practice effect. However, we found no evidence of any difference in performance metrics between groups who viewed 2D versus 3D television. We conclude that there is no evidence that viewing S3D television produces acute impairments in balance or coordination.

Accelerometry did reveal a difference between 2D and 3D TV in the younger age-groups (under 24). The variability in acceleration measurements increased in the youngest age-group after viewing S3D (but not 2D) TV. However, the significance in the ANOVA analysis was largely driven by two child participants in the active 3D group, who reported eyestrain/nausea and who showed some of the largest increases in variability. One of these two children had pre-existing problems with binocular alignment, and the other removed his 3D glasses during viewing, perhaps because the adult-sized active 3D glasses were heavy and uncomfortable. Given that the effect of TV-group disappeared when these two participants were removed, it is difficult to assess its significance. It is certainly not compelling evidence for an effect of S3D TV.

Our conclusions are necessarily limited in scope. First, participants generally waited a few minutes after the end of the movie before carrying out the balance and coordination tests for the second time. Participants watched the movie in groups of up to 5, and the tests took around 2 min to complete, so some participants would have waited as long as 10 min before being tested. Thus, our study does not rule out very short-lived impairments which decay away over this time scale. On the other hand, such short-lived effects are unlikely to be problematic in everyday life, since they would have vanished by the time someone had walked out to the parking lot from the cinema, for example. Conversely, this study examined only effects after a one-time viewing of a S3D movie, i.e. around 80 min of S3D content. It therefore does not examine any longer term effects which could build up over repeated exposure to S3D.

An unavoidable limitation is simply that any impairment may have been too subtle, or affect too few viewers, for us to pick up. We did not find any evidence of impairment; it is not the case that we found a trend which failed to reach statistical significance. Nevertheless, it remains possible that 3D does cause a visuomotor impairment in some people, but that we did not detect this. Any impairment would have to be slight, since our methods were demonstrably powerful enough to pick up some fairly subtle effects. For example, we successfully detected an effect of age on time taken to complete the balance task, and our accelerometry detected highly significant effects of age and task, i.e. we could detect the differences in body motion between the ramp, beam and steps tasks. Thus, our null results for TV-group indicate that any hypothetical impairment must be small and/or affect very few people. Table 2 quantifies the upper bounds which our study places on various potential effects.

In general, adverse effects reported with S3D are highly content-specific; for example, the amount of visual fatigue depends strongly on factors such as the magnitude or rate of change of vergence/accommodation conflict [8,30]. One would similarly expect that any short-term impairment in balance or coordination would also depend on the specific content, for example the magnitude of large-field self-motion cues. Thus, strictly, our study shows only that the particular content we examined does not cause measurable impairments. A distinctive feature of ‘Toy Story’ is that it is entirely computer-generated imagery (CGI). This choice was dictated by practicalities: we wanted a popular feature-length movie available in 2D and S3D, which would be engaging and not upsetting for viewers of all ages including small children, and this effectively restricted us to animated movies. This may restrict the general validity of our results. In CGI, it is easier to control some of the problematic features of S3D. For example, scenes can be rendered using different camera geometry for foreground versus background, in order to allow foreground objects to appear fully rounded without introducing excessive disparities in the background. Thus, the results we obtained with this carefully crafted Pixar movie may not hold for live-action S3D football. An alternative approach would be to deliberately design laboratory S3D content so as to maximize the probability of adverse effects (large-field cues to self-motion, large vergence/accommodation conflict, rapid parallax changes, etc.) and examine whether that can produce measurable changes in balance and coordination. In this initial study, we felt it was more important to see whether a representative commercial S3D movie caused impairment.

Another limitation is that we have examined our chosen content only viewed on a television screen at a distance of 2.5 m, not in other settings, e.g. a cinema or mobile device. We have found previously that viewers report more adverse effects with 3D television and video games than they do with 3D cinema [7]. This may be because the shorter viewing distance exacerbates the vergence/accommodation conflict [1–3,8,10,25,27,35]. Thus, it seems unlikely that S3D cinema would cause detectable impairments of balance and coordination, given the lack of effect of S3D TV. On the other hand, we cannot exclude the possibility that detectable impairments could occur after viewing S3D content at shorter viewing distance, e.g. on a 3D smartphone.

Given that this is the first study to attempt to identify objective motor impairments associated with viewing S3D, and given the increasingly widespread use of 3D, the fact that we could not detect any measurable impairment is welcome reassurance.

## Supplementary Material

LABSTUDY_COMPLETE_v8h = complete data in Matlab format. Other files ending .m: Matlab files needed to create the data figures.

## Data accessibility

The datasets supporting this article have been uploaded as part of the electronic supplementary material, along with Matlab code necessary to reproduce the data figures.
